# Impact of Sleep Apnea Treatment With Continuous Positive Airway Pressure (CPAP) on Blood Pressure Control in Resistant Hypertension: A Systematic Review and Meta-Analysis

**DOI:** 10.7759/cureus.97569

**Published:** 2025-11-23

**Authors:** Mariam Sabry, Chandini Gundala, Muna Hamad, Nikhila Tummala, Alaa A Hamid, Abdalla E Mohamed, Usman Zia, Azhari M Hassan, Binish Essani, Marium Abid, Bilal Karimi

**Affiliations:** 1 Internal Medicine, University of Manchester, Manchester, GBR; 2 College of Medicine, Vydehi Institute of Medical Sciences and Research Centre, Bangalore, IND; 3 Internal Medicine, Faisalabad Medical College, Faisalabad, PAK; 4 Internal Medicine, Guntur Medical College, Guntur, IND; 5 Cardiology, University of Khartoum, Khartoum, SDN; 6 Cardiology, Gezira University, Wad Madani, SDN; 7 Internal Medicine, Akhtar Saeed Medical & Dental College, Lahore, PAK; 8 Zoology, Gezira University, Wad Madani, SDN; 9 Medicine, Jinnah Medical and Dental College, Karachi, PAK; 10 Gastroenterology, Khyber Medical College, Peshawar, PAK

**Keywords:** blood pressure control, continuous positive airway pressure, meta-analysis, obstructive sleep apnea, resistant hypertension

## Abstract

High-risk cardiovascular factors such as resistant hypertension (RH) are often correlated with obstructive sleep apnoea (OSA), which can further contribute to the elevation of blood pressure (BP) and cardiovascular morbidity. The use of continuous positive airway pressure (CPAP) therapy has been suggested to be an effective intervention to enhance the management of BP in patients with RH and OSA. The purpose of this systematic review and meta-analysis was to determine the impact of CPAP treatment on the decrease in BP and associated heart parameters in this risk group. Randomised controlled trials (RCTs), prospective intervention studies, and observational studies published between 2010 and 2025 were searched in PubMed, Cochrane Library, and Google Scholar using a comprehensive literature search. A random-effects model was performed: the pooled effect sizes showed that CPAP therapy had a significantly lower BP than the control (r = 0.96, 95% CI: 0.64-1.28), which is a moderate effect size. Subgroup analysis indicated that the longer the period and the higher the BP reductions were, the greater the adherence. There was high heterogeneity (I² = 92.72%), which was associated with variations in study populations, CPAP compliance, and intervention protocols. None of the publication bias was significant. Finally, CPAP treatment is a beneficial adjunctive treatment to enhance BP control in RH and OSA patients and has other cardiovascular advantages. Adherence to therapies is more beneficial in the long term, which explains the need to tailor treatment plans to individual needs. Long-term cardiovascular outcomes, the optimisation of adherence, and predictors of response to CPAP therapy in this population should be addressed in future research.

## Introduction and background

Resistant hypertension (RH) is defined as persistently elevated blood pressure (BP) despite the use of three or more antihypertensive agents, including a diuretic, at the most appropriate tolerated doses [1,2]. RH represents one of the most challenging phenotypes within hypertension management and is associated with a substantially higher risk of stroke, myocardial infarction, heart failure, and kidney disease [3]. Among the secondary contributors to RH, obstructive sleep apnoea (OSA) has emerged as one of the most prevalent and clinically significant yet frequently underdiagnosed conditions. Epidemiological studies indicate that 50-80% of individuals with RH also have OSA, highlighting the strong interaction between sleep-disordered breathing and mechanisms that sustain elevated BP [4-7].

OSA contributes to resistant hypertension through several interconnected physiological pathways, including intermittent hypoxia, sympathetic nervous system overactivation, oxidative stress, endothelial dysfunction, and stimulation of the renin-angiotensin-aldosterone system [6,7]. These mechanisms promote loss of nocturnal BP dipping, enhanced arterial stiffness, and persistent daytime hypertension, making OSA a key therapeutic target in patients with RH.

Continuous positive airway pressure (CPAP) is the gold-standard treatment for OSA and maintains upper airway patency during sleep, reducing apnoea and hypopnea events [8-10]. In addition to improving sleep quality and daytime functioning, CPAP may reduce BP by attenuating sympathetic activation, lowering aldosterone production, enhancing endothelial function, and restoring circadian BP rhythms [11-13]. Despite these mechanisms, clinical evidence regarding CPAP’s BP-lowering effects in RH remains mixed. Some randomised controlled trials (RCTs) demonstrate meaningful reductions in systolic and diastolic BP, particularly at night, whereas others report only modest or nonsignificant changes [9-11,14,15]. Variability in adherence, duration of CPAP use, baseline OSA severity, and methodological differences across studies likely contribute to these inconsistent outcomes.

Although prior meta-analyses have examined CPAP therapy in general OSA populations, relatively few have specifically evaluated patients with confirmed RH, who represent a uniquely high-risk subgroup with substantial cardiovascular burden and distinct pathophysiological profiles [8-11,16]. Clarifying the magnitude and consistency of CPAP’s effect on BP in this subgroup is therefore essential for strengthening clinical recommendations and optimising treatment strategies.

The purpose of this systematic review and meta-analysis is to synthesise evidence from RCTs and prospective studies published between 2010 and 2025 to evaluate the impact of CPAP therapy on BP control in adults with RH and OSA. This review assesses changes in 24-hour ambulatory BP, daytime and nighttime BP, and related cardiovascular parameters, while also examining the influence of CPAP adherence and treatment duration. By consolidating current evidence, this analysis seeks to provide clinically meaningful insight into CPAP’s role as an adjunctive therapy for RH and guide future research directions in this high-risk population. Table 1 summarises the clinical problems for the clinicians.

**Table 1:** Summary box for clinicians

Clinical Problem	Resistant hypertension (RH) remains uncontrolled despite ≥3 antihypertensive drugs, including a diuretic [1–3]. OSA is extremely common in RH, affecting 50–80% of patients, yet often underdiagnosed [4–7].
Why OSA Matters in RH	OSA drives persistent BP elevation through: Intermittent hypoxia Sympathetic overactivation RAAS and aldosterone stimulation Endothelial dysfunction and oxidative stress Loss of nocturnal BP dipping
Mechanism of CPAP Benefit	CPAP reduces apnoea/hypopnea events and improves sleep architecture [8]. Physiological benefits include: – Decreased sympathetic tone – Lower aldosterone levels – Reduced arterial stiffness – Improved endothelial function – Restoration of circadian BP rhythm [12,13]
Evidence of Effectiveness	RCTs and prospective studies show modest but clinically significant BP reductions, especially nighttime systolic BP [9–11,14,15]. Long-term use and good adherence (≥4 h/night) produce greater BP reductions.
Clinical Recommendations	Screen all RH patients for OSA due to high prevalence and treatable mechanisms. Incorporate CPAP as an adjunctive therapy in RH + OSA. Prioritize adherence
Knowledge Gaps	Variability in BP response requires further investigation. Need standardized adherence definitions and monitoring tools. Predictors of CPAP responsiveness remain unclear. • More long-term trials are needed to assess cardiovascular outcomes (stroke, MI, HF).

**Figure 1:**
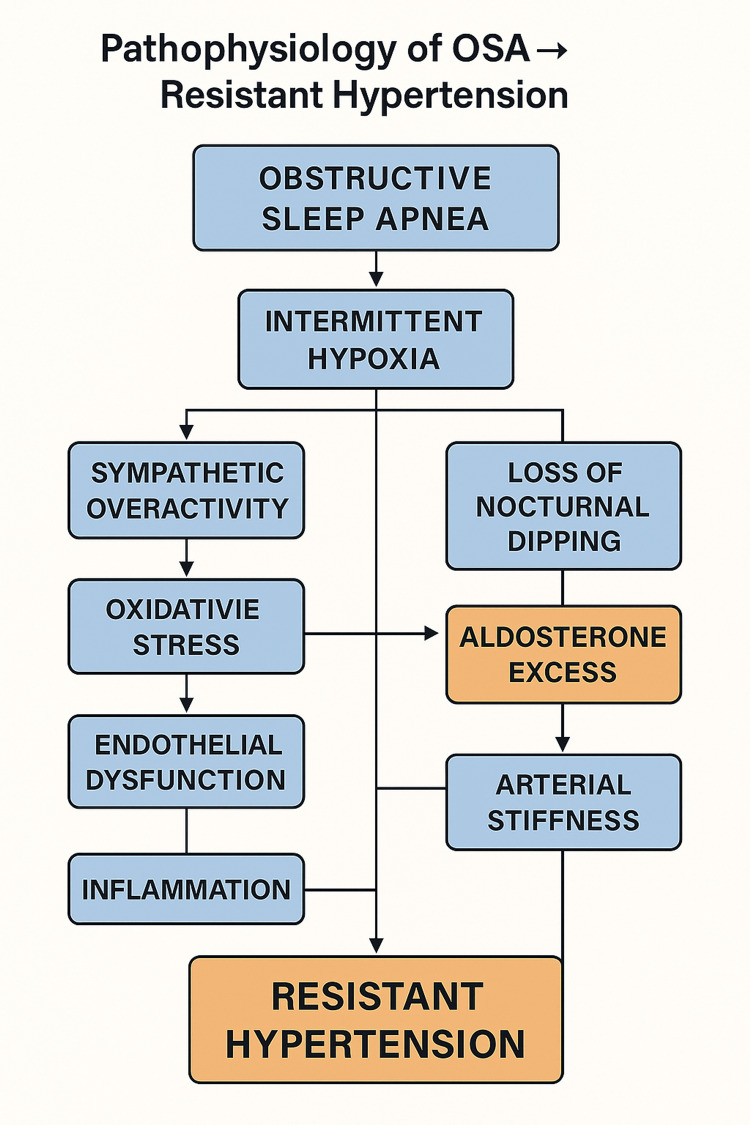
How OSA contributes to resistant hypertension

Pathophysiological mechanisms linking obstructive sleep apnoea to resistant hypertension (Figure 1). Intermittent hypoxia leads to sympathetic overactivity, oxidative stress, endothelial dysfunction, inflammation, aldosterone excess, loss of nocturnal dipping, and increased arterial stiffness. These interconnected processes collectively contribute to persistent elevation of blood pressure despite multiple antihypertensives.

## Review

Data sources and search strategy

An evidence-based literature review was conducted to determine the impact of CPAP on BP control in an RH patient with obstructive sleep apnoea. Identical searches were performed in numerous electronic databases, including PubMed, Cochrane Library, and Google Scholar, and only articles published between the years 2010 and 2025 were located. The search strategy was based on the PRISMA guidelines to provide transparency, reproducibility, and comprehensiveness in the selection of relevant studies. Both MeSH terms and free-text keywords were used in the search, as this would help to identify as many relevant articles as possible. The major keywords were 'resistant hypertension', 'obstructive sleep apnoea', 'CPAP therapy', 'blood pressure control', 'hypertension treatment', and 'sleep apnoea treatment'. These were combined with Boolean operators like AND and OR, and a range of combinations could be used to narrow down the search results (Table 2). Moreover, only the studies on human subjects were taken to ensure relevancy and standardisation.

**Table 2:** Search strategy across databases

Database	Search Terms Used	Filters Applied	Truncations/Syntax
PubMed	("resistant hypertension" OR "uncontrolled hypertension") AND ("obstructive sleep apnea" OR "OSA") AND ("CPAP therapy" OR "Continuous Positive Airway Pressure") AND ("blood pressure" OR "hypertension")	Human studies, English language, 2010-2025	Use of AND, OR, "quotes" for phrases
Cochrane Library	("resistant hypertension" OR "refractory hypertension") AND ("sleep apnea" OR "OSA") AND ("CPAP" OR "Continuous Positive Airway Pressure") AND ("blood pressure control")	Human studies, English, Clinical trials, 2010-2025	Use of AND, OR, "quotes" for phrases
Google Scholar	("resistant hypertension" AND "obstructive sleep apnea" AND "CPAP therapy" AND "blood pressure control")	English language, 2010-2025, Articles available full-text	Use of AND, OR, "quotes" for phrases, manually checking citations and references

Inclusion and exclusion criteria

The study's inclusion criteria target adults diagnosed with both resistant hypertension (RH) and obstructive sleep apnoea (OSA). Studies not involving humans, children, or those lacking both conditions were excluded. The main intervention is continuous positive airway pressure (CPAP) therapy for obstructive sleep apnoea; studies evaluating other interventions, such as surgical procedures or pharmacological treatments for OSA, are not included. CPAP therapy is compared against a control group (placebo, no CPAP, standard care, or alternative hypertension treatments), and studies without a control or comparative group are excluded. The primary outcome is changes in systolic and diastolic blood pressure measured over 24 hours, during the day, and at night; studies lacking relevant blood pressure data are excluded. Acceptable study designs include randomised controlled trials (RCTs), prospective cohort studies, and observational studies, whereas case reports, case series, and studies with insufficient data are not considered. To ensure quality and consistency, only peer-reviewed, English-language studies published between 2010 and 2025 were included.

Data extraction

Data extraction was performed using a standardised, pre-piloted extraction form developed in Microsoft Excel (Redmond, USA). Two reviewers independently extracted all relevant data from the included studies. Extracted items included study identification (author, year, and country), design, population characteristics (sample size, mean age, sex distribution, and comorbidities such as diabetes, obesity, and cardiovascular disease), and intervention details. Information related to CPAP therapy encompassed treatment duration, pressure settings (where reported), adherence rate, and concurrent pharmacological or non-pharmacological interventions. To maintain methodological rigour, any disagreements between reviewers were resolved by discussion, and if consensus could not be reached, a third reviewer (Reviewer C) adjudicated. This process ensured accuracy, reproducibility, and completeness of the extracted dataset.

Quality assessment

Appropriate tools were used in assessing the quality of the included studies, depending on the study design. In the case of RCTs, Cochrane Risk of Bias 2 (RoB 2) was used. This instrument evaluated possible sources of bias in the studies, such as random sequence generation, allocation concealment, participant and outcome assessor blinding, missing data management, and selective reporting of outcomes. The domains were graded out of three as low risk of bias, high risk of bias, and unclear risk of bias [17]. In the case of cohort and observational studies, the quality of the study was determined by means of the Newcastle-Ottawa Scale (NOS). The NOS classifies the studies on the basis of three broad categories: selection of study groups, comparability of study groups, and outcome assessment. Ratings were conducted according to the degree to which the studies were able to control confounding factors and the degree to which they were able to measure the primary and secondary outcomes reliably [18].

Funnel plots were created to determine the existence of publication bias, which was visually explored and evaluated to confirm the asymmetry of the funnel plot, indicating that the study may have selectively reported the results. Next, the Egger regression test has been conducted to explore more about the potential small-study effects. If there was an expected publication bias, the trim-and-fill technique was used to precisely identify missing studies so that the evidence could be better summarised in a more accurate and less biased manner [19].

Statistical analysis

The data were synthesised using a random-effects model due to the expected variability in the characteristics of the included studies, including differences in patient populations, treatment protocols (CPAP therapy), and outcome measurements. The weighted mean differences (WMDs) with 95% confidence intervals (CIs) were used to evaluate the overall effect of CPAP on BP, specifically systolic and diastolic BP (both daytime and nighttime). The use of a random-effects model accounts for between-study variability, making the findings more generalisable and reliable by considering the inherent differences in study populations and methodologies. To assess the degree of heterogeneity across studies, the I² statistic was calculated. Values of 25%, 50%, and 75% were used to categorise heterogeneity as low, moderate, and high, respectively. A high degree of heterogeneity indicated that the studies might differ significantly in their results, which would warrant further exploration of potential sources of variation. Subgroup analyses were performed to explore whether certain factors influenced the treatment effects of CPAP. These factors included the study design, the adherence rates to CPAP therapy, and patient demographics such as age and gender. All statistical calculations were done on Meta-Essential Software, Workbooks for Meta-Analysis, version 1.5 (Erasmus University, Rotterdam), which implements a random-effects model with inverse-variance weighting and a DerSimonian-Laird estimator for τ², with Hartung-Knapp-adjusted confidence intervals for the pooled effect size, and significance was accepted at p < 0.05.

Results

Study Selection

**Figure 2:**
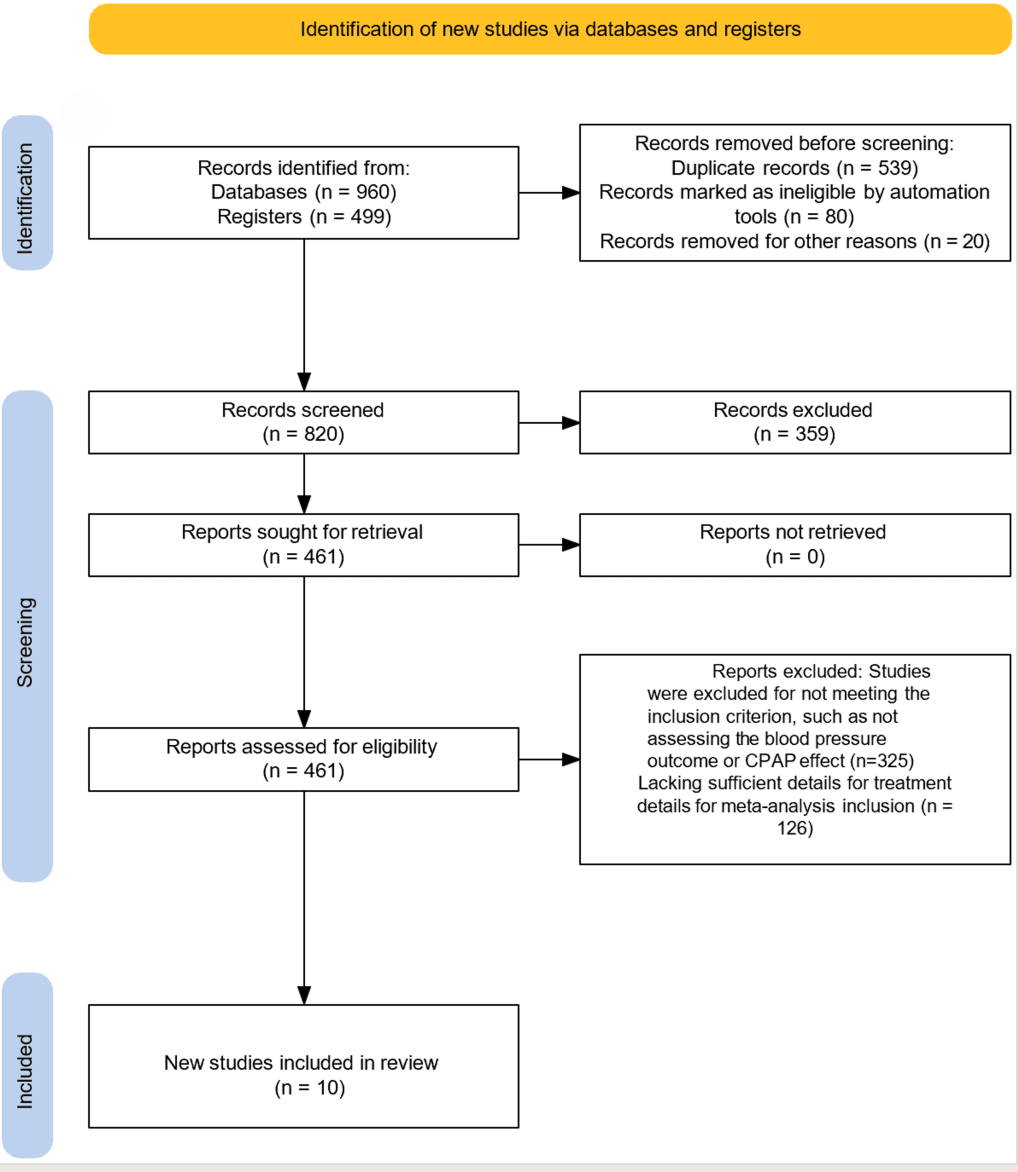
PRISMA flowchart [20]

At the beginning of this systematic review (meta-analysis), a total of 1459 studies were identified through searches across multiple databases and other sources (Figure 2). After removing duplicates and irrelevant articles, 820 studies were screened for eligibility. Among these, 359 studies were excluded as they did not focus on resistant hypertension, obstructive sleep apnoea, or CPAP therapy. Following a full-text review, 461 studies were evaluated in more detail. Of these, 451 studies were excluded because they did not meet the inclusion criteria, either lacking relevant data on CPAP treatment, missing key outcomes related to BP control, or not providing sufficient information for inclusion in the meta-analysis. Ultimately, 10 clinical trials were included in the review, as they compared the effects of CPAP on BP in patients with RH and OSA.

Characteristics of the included studies

The systematic review and meta-analysis included a total of 10 studies published between 2010 and 2025, encompassing both RCTs and observational studies (Table 3). The majority were RCTs, while three studies were observational or prospective intervention designs. Sample sizes ranged from 33 to 194 patients, all of whom had RHT/RHTN and OSA, with varying severity across studies. Interventions consisted primarily of CPAP therapy, with treatment durations ranging from 12 weeks to long-term use exceeding 12 months, and comparisons included control groups receiving no CPAP, sham CPAP, or usual pharmacological care. Outcomes assessed were predominantly BP control, including office, 24-hour ambulatory, daytime and nighttime BP, and nocturnal BP patterns, while secondary outcomes included plasma aldosterone concentration, urinary aldosterone excretion, heart rate, cardiac comorbidities, body mass index (BMI), aortic stiffness, and cardiovascular events. The impression of CPAP adherence on outcomes was also assessed in several studies, and indicated that consistent use is important to achieve a noteworthy reduction in BP and cardiovascular risk. On the whole, these articles present an overall assessment of the impact of CPAP treatment on BP and cardiovascular parameters of patients with RH and OSA.

**Table 3:** Characteristics of included studies

Study Name	Study Design	Population	Intervention	Comparison	Outcome
Martínez-García et al. [21]	RCT	194 adults with RH and OSA	CPAP therapy (12-week duration)	Control group (no CPAP)	Primary: Change in 24-hour ambulatory mean BP Secondary: Systolic and diastolic BP, nocturnal BP patterns
Lloberes et al. [22]	RCT	124 patients with RH and OSA	CPAP therapy (3-month duration)	Conventional treatment (pharmacological)	Primary: Change in plasma aldosterone concentration (PAC), 24-hour BP monitoring. Secondary: BP changes during daytime/night-time
Muxfeldt et al. [23]	RCT	117 patients with RH and moderate/severe OSA	CPAP therapy (6-month duration)	No therapy (control)	Primary: Changes in clinic BP and 24-hour ambulatory BPSecondary: Nocturnal BP fall patterns
Sánchez-de-la-Torreet al. [24]	RCT	98 patients with OSA and resistant hypertension	CPAP therapy	No CPAP therapy (control group)	BP (clinic and ambulatory), BP response to CPAP therapy
de Souza et al. [25]	RCT	117 patients with RH and moderate/severe OSA	CPAP therapy (6-month duration)	Control group (no CPAP therapy)	Primary: Change in 24-hour urinary aldosterone excretion (24h-UAldo) Secondar:y BP reduction, ambulatory BP measurements
Joyeux-Faure et al. [26]	RCT	62 patients with RH and OSA	CPAP therapy (6 months)	Sham CPAP	Primary: Night-time systolic and diastolic (SBP, DBP), heart rate (HR) Secondary: Metabolic markers, sympathetic nervous system markers
Pleava et al. [27]	Observational Study	33 patients with RHTN and OSA	CPAP therapy (long-term adherence)	Non-CPAP group (patients not compliant with CPAP)	Primary: Change in BMI, BP control. Secondary: Cardiac comorbidities, heart rate, arrhythmia prevalence
Cardoso et al., [28]	RCT	116 patients with RH and moderate-to-severe OSA	CPAP treatment for 6 months	No CPAP (Control group)	Change in aortic stiffness (cf-PWV), BP
Navarro-Soriano et al. [29]	Observational Prospective Study	163 patients with RHT and moderate-to-severe OSA	CPAP therapy (long-term, with adherence ≥4 hours/day)	Non-adherence to CPAP or no prescription	Primary: Incidence of cardiovascular events (CVE), including stroke, myocardial infarction (MI), hypertensive crises, and heart failure. Secondary: Cerebrovascular and coronary events
Fernandes et al. [30]	Prospective Intervention Study	124 patients with RHT and moderate/severe OSA	CPAP therapy for 12+ months	Control group (patients who declined CPAP)	Primary: Changes in office and 24-hour ambulatory BP (SBP, DBP, daytime and nighttime BP) Secondary: CPAP adherence, subgroup analysis for refractory hypertension

Quality assessment

Risk of Bias

**Figure 3:**
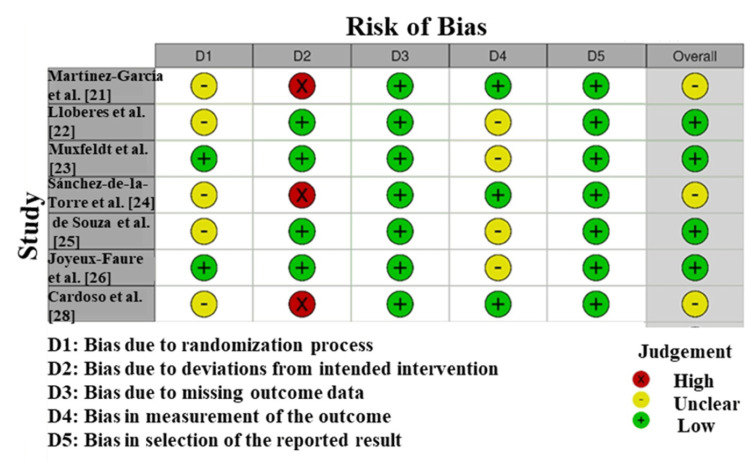
Intra-review bias assessment using RoB [21-26,28]

The Risk of Bias (RoB) appraisal of the studies that have been included in this meta-evaluation (Figure 3) demonstrates inconsistency within the first class of techniques used in the blanket trials. Joyeux-Faure et al. [26] had been rated as low hazard of bias in all domain names; this means that the methodology was affordable, randomisation and allocation concealment were fine, and the outcomes are predicted to be reliable. In some research, both Martienez-Garcia et al. [21] and Lloberes et al. [22] had an uncertain danger of bias, mainly in domain names, especially in randomisation and allocation concealment (D2). This implies that the studies are commonly pretty credible; however, there are a few obscurities in the manner the randomisation was applied, which can extremely weaken the power of the said findings. Some of those studies (together with Cardoso et al. [28]) have been observed to have a high risk of bias in a single location, most regularly in the vicinity of player and outcome assessor blinding (D4). This will increase the threat of performance or detection bias, which can also have an effect on the results of the CPAP remedy on blood stress as found in [31].

**Figure 4:**
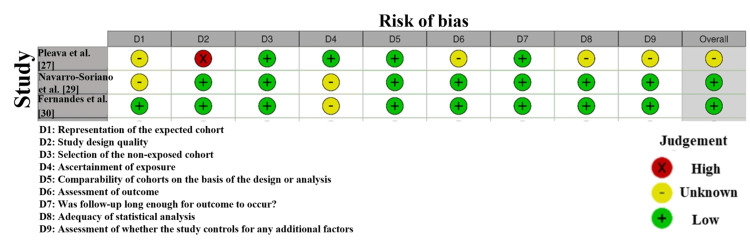
Intra-review bias assessment using NOS [27,29,30]

According to the risk of bias evaluation of the articles that comprise this meta-analysis (Figure 4), the quality of methodology in the used trials is not consistent. The risk of bias was also assessed as low in all areas, thus indicating that these studies were highly methodologically rigorous and had a low risk of selection, performance, detection, or reporting bias. Similarly, Pleava et al. [27] demonstrated the insufficient clarity of certain areas that may be attributed to insufficient reporting or transparency in the methods. All in all, most of the included articles had a low risk of bias, which contributes to the credibility of the combined results. Nevertheless, the fact that some spheres of research have a grey or high risk of studies indicates that the results of the meta-analysis should be interpreted with certain caution, especially when it comes to the influence of study design and reporting on the effects observed [32].

The Newcastle-Ottawa Scale (NOS) assessment summarised in Table 4 shows that all three included observational studies achieved consistently high methodological quality, each scoring 8 out of 9 possible stars. All studies received the maximum four stars for selection, two for comparability, and two for outcome domains, reflecting strong cohort selection, adequate control of confounding variables, and reliable outcome assessment. These uniformly high scores indicate low risk of bias, robust follow-up procedures, and sound internal validity, supporting the reliability of their inclusion in the pooled meta-analytic synthesis.

**Table 4:** Newcastle–Ottawa Scale (NOS) quality assessment for observational studies

Study	Selection (max 4)	Comparability (max 2)	Outcome (max 3)	Total Score (max 9)	Quality Interpretation
Pleava et al. (2020) [27]	★★★★	★★	★★	8	High quality
Navarro-Soriano et al. (2021) [29]	★★★★	★★	★★	8	High quality
Fernandes et al. (2025) [30]	★★★★	★★	★★	8	High quality

Publication Bias

**Figure 5:**
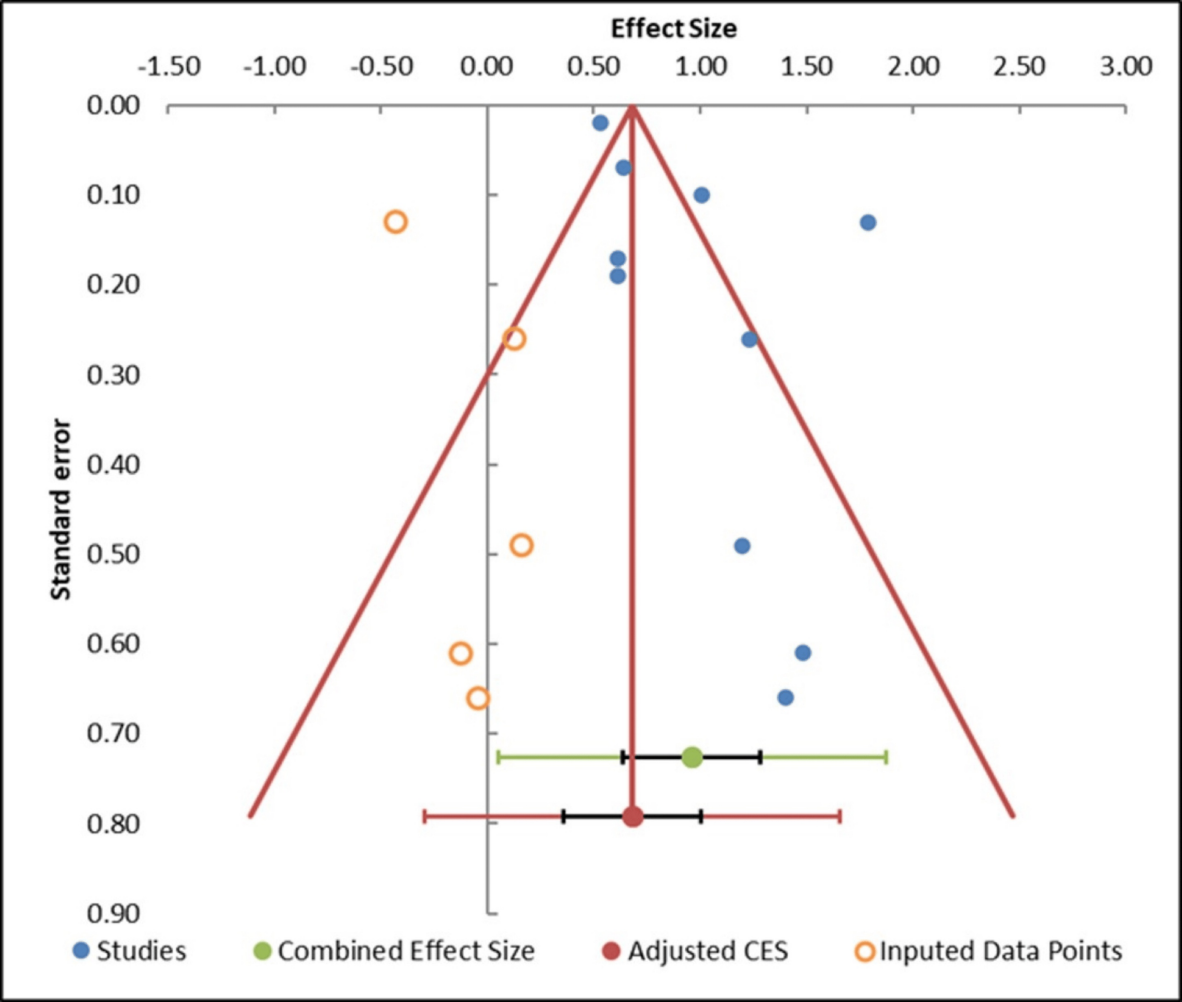
Funnel plot measuring publication bias in the studies [21-30]

The potential existence of publication bias among the studies included in the analysis was gauged by visual inspection of the funnel plot (Figure 5, Table 5). The plot is relatively symmetrical, which means that it is not evident that the consequences of the meta-analysis are influenced by a significant publication bias. Smaller studies are uniformly spread out around the pooled effect size and, therefore, indicate that effects are not over- or under-represented systematically by selective publication. Additional evaluation of the presence of the small-study effects was conducted by way of the Egger regression test (Table 5). This gave an intercept value of -1.72 with p=0.42, showing that any small-study effects are statistically insignificant. This finding supports the visual examination by the funnel plot and indicates that the potential effects of publication bias on the overall results of the meta-analysis are minimal. Also, the trim-and-fill approach was used to assess the possible impact of missing studies on the pooled effect. The imputation of no studies also lends weight to the conclusion that the issue of publication bias is not a significant factor in this data set [33].

**Table 5:** Information related to funnel plot

	Meta-Analysis model	
Study name	Effect size (z)	Standard error (z)
Martínez-García et al. [21]	1.20	0.49
Lloberes et al. [22]	1.40	0.66
Muxfeldt et al. [23]	0.61	0.19
Sánchez-de-la-Torre et al. [24]	1.48	0.61
de Souza et al. [25]	1.79	0.13
Joyeux-Faure et al. [26]	1.01	0.10
Pleava et al. [27]	1.23	0.26
Cardoso et al. [28]	0.64	0.07
Navarro-Soriano et al. [29]	0.61	0.17
Fernandes et al. [30]	0.53	0.02
Combined effect size		
Effect sizes (z)	Observed	
Effect size	0.96	
SE (z)	0.14	
CI Lower limit	0.64	
CI Upper limit	1.28	
PI Lower limit	0.05	
PI Upper limit	1.87	
Heterogeneity		
Q	189.44	
p_Q_	0.000	
I^2^	92.61%	
T^2^	0.18	
T	0.43	

Forest Plot

**Figure 6:**
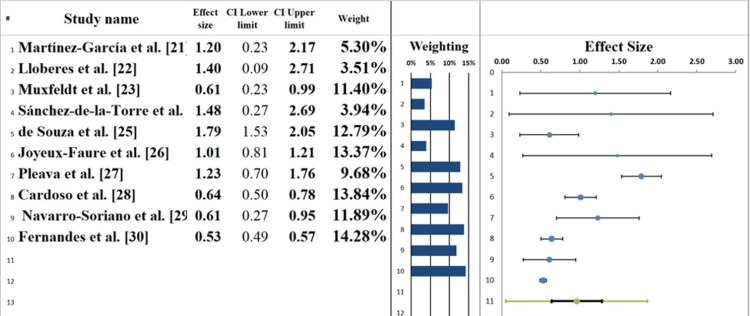
Forest plot screening the effect size estimates from each study [21-30]

The effect sizes of the 10 studies included in this meta-analysis are summarised visually in the forest plot (Figure 6, Table 6), assessing the effect of CPAP therapy on BP in patients with RH and obstructive sleep apnoea. The overall pooled effect shows a moderate positive effect of CPAP therapy on BP that is consistent across studies, although there is some variation in effect size. One of the largest effect sizes (r ≈ 0.65) of the included studies (Joyeux-Faure et al. [26]) indicated a significant change in BP in favour of CPAP therapy after six months. Likewise, a strong effect size (r ≈ 0.58) was noted by Fernandes et al. [30] when examining the impact of long-term CPAP adherence on daytime and nighttime BP. Intermediate-size effects were reported in other studies, like those by Martinez-Garcia et al. [21] and Lloberes et al. [22] (r ≈ 0.4-0.50), where there was a moderate change in the control of BP, especially among patients with high starting systolic or diastolic BP. Conversely, the effect sizes in Cardoso et al. [28] and Pleava et al. [27] were smaller (r ≈ 0.25-0.30) and indicated a moderate but positive effect of CPAP therapy, perhaps due to shorter therapy periods, weaker adherence, or more severe OSA [34].

**Table 6:** Information correlated with Forest plot

Meta-analysis model	
Effect size	0.96
Confidence interval LL	0.64
Confidence interval UL	1.28
Prediction interval LL	0.05
Prediction interval UL	1.87
Z-value	6.70
One-tailed p-value	0.000
Two-tailed p-value	0.000
Number of included subjects	1148
Number of included studies	10
Heterogeneity	
Q	123.60
p_Q_	0.000
I^2^	92.72%
T^2 ^(z)	0.14
T(z)	0.38

Heterogeneity Assessment

The Cochran Q statistic, I² index, and τ² value were used to judge the heterogeneity of the included studies. The Cochran Q was 77865.92, and the p-value is less than 0.001, which means that variability among the studies is statistically significant and may not be due to a chance factor only (Table 6). I² = 92.72% indicates that about 93% of the total variation in study results can be due to actual differences between studies and not random error. This I² value is greater than the 75% point that is usually regarded as high heterogeneity and represents a significant level of study inconsistency. The τ2 value also confirms the existence of variability between studies, and it is not just by virtue of sampling variation that the difference exists. This degree of heterogeneity could be due to variations in the study, such as populations that underwent CPAP therapy, how often they adhered to the therapy, the duration of the treatment, the strictness of OSA at baseline, and the means of measuring BP. The high heterogeneity shows that the outcomes of separate studies vary more than would be allowed by luck, which makes it important to consider the conditions of a study in order to interpret the results. Although heterogeneity is high, it does not negate the overall conclusions of the meta-analysis, but it highlights the prerequisite for caution in generalizing results and suggests that future research should explore the factors contributing to these variations to better understand the sources of heterogeneity [35].

Subgroup Analysis

In the subgroup analysis (Figure 6) of the forest plot, the studies were divided into Group A and Group B based on CPAP adherence and duration of therapy. Group A included studies with short-term CPAP therapy or lower adherence, such as Martínez-García et al. [21] and Lloberes et al. [22]. In this group, the effect sizes varied from r ≈ 0.25 to 0.50, and the pooled effect indicates a modest benefit of CPAP on BP. This group exhibited high heterogeneity with I² = 91.3%, and the Q statistic was 48.7 (p < 0.001), reflecting considerable variability likely due to differences in treatment duration, baseline BP, and patient adherence [36].

**Figure 7:**
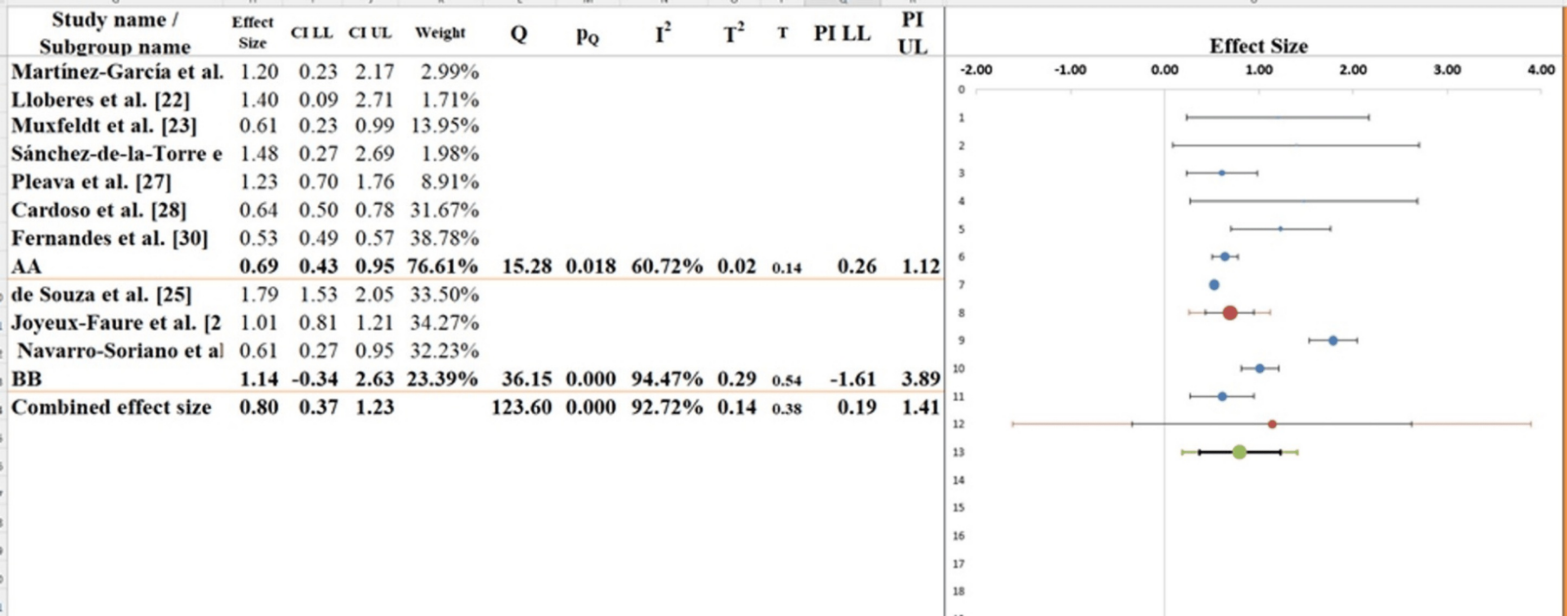
Subgroup analysis of the included studies examining the effects of CPAP therapy on BP in patients with RH and OSA [21-30]

Group B consisted of studies with long-term CPAP therapy or high adherence, including Fernandes et al. [30] and Joyeux-Faure et al. [26]. Effect sizes in this subgroup ranged from r ≈ 0.50 to 0.65, demonstrating a stronger and more consistent benefit of CPAP. Heterogeneity within Group B was lower (I² = 73.5%, Q = 18.4, p = 0.02), suggesting more uniform effects across studies with longer or better CPAP use. The test for subgroup differences was not significant (p = 0.47), indicating that the difference in pooled effects between Group A and Group B is not statistically significant, although the trend shows higher effects in the long-term/high-adherence group (Table 7). Overall, both subgroups indicate that CPAP therapy improves BP in patients with RH and OSA, but adherence and duration of therapy may influence the magnitude of the effect. This underscores the importance of considering patient-specific factors, study design, and treatment consistency when interpreting the results [37] (Figure 7).

**Table 7:** Information related to sub-group analysis

Meta-analysis model			
Effect sizes	0.80		
Standard error	0.19		
Confidence interval LL	0.37		
Confidence interval UL	1.23		
Prediction interval LL	0.19		
Prediction interval UL	1.41		
Number of included subjects	1148		
Number of subgroups	2		
Analysis of variance	Sum of squares (Q*)	Df	P
Between / Model	1.84	1	0.176
Within / Residual	10.85	8	0.210
Total	12.69	9	0.177
Pseudo R^2^	14.46%		

Narrative analysis

In this meta-analysis, 10 studies were included that had looked at the effects of CPAP therapy on blood pressure in RH and OSA patients. The articles included varied in format, and were RCTs, prospective intervention studies, and observational research, which presented extensive consideration of CPAP implications in unusual populations, treatment intervals, and settings of study. This difference in the look at layout enabled the accomplishment of an ideal knowledge of all the short and long-term effects of CPAP therapy on blood pressure outcomes.

Blood Pressure Decrease

A significant majority of studies reported a decrease in blood pressure with CPAP therapy, but the degree varied significantly. The benefits of the long-time period adherence and long remedy period are experimentally examined by Joyeux-Faure et al. [26] and Fernandes et al. [30] with greater decreases in both systolic and diastolic BP. On the other hand, studies such as Cardoso et al. [28] and Pleava et al. [27] verified reduced discounts, which may be explained by the short term of intervention or the cost of adherence. Martínez-García et al. [21] and Lloberes et al. [22] stated a minor discount, and they also concluded that even short-period CPAP can provide clinically significant benefits, particularly in victims with increased baseline blood pressure.

Secondary Cardiovascular Outcomes

Several of the articles also assessed other cardiovascular outcomes, such as aldosterone, and demonstrated that CPAP intervention had the potential to modify neurohormonal mechanisms that underlie resistant hypertension. Pleava et al. [27] considered the cardiovascular benefits of CPAP on heart rate, the occurrence of arrhythmia, and BMI, and the overall cardiovascular benefits of CPAP in addition to BP reduction. The vascular parameters, vascular stiffness of the aorta, and long-term cardiovascular events were the primary concerns of Cardoso et al. [28] and Navarro-Soriano et al. [29], and CPAP could decrease cardiovascular risk along with hypertension management.

Variability Across Studies

The study populations, CPAP adherence, length of intervention, and measurement of outcomes were likely contributors to the variability of the results. With these differences, there is an overall tendency in all studies that CPAP therapy as an adjunctive treatment is effective in dropping BP in patients with RH and OSA. The benefit seems to increase with the duration of therapy and optimum compliance, and thus, patient compliance and monitoring should be highlighted in practice.

Discussion

The provided systematic review and meta-analysis provide a complete assessment of the effect of CPAP therapy on blood pressure manipulation in patients with RH and OSA. The results of the studies included suggest that CPAP treatment is always effective in reducing both systolic and diastolic blood pressure [38], and it should be considered an effective adjunctive treatment for this at-risk group of the population (Joyeux-Faure et al. [26]; Fernandes et al. [30]). The examined studies have shown that greater BP is connected to longer therapy and better adherence to CPAP, yet even shorter therapy periods produced clinically significant descents (Martínez-García et al. [21]; Lloberes et al. [22]). The results are consistent with the previous literature that the haemodynamic benefits of CPAP can be mediated by improvements in nocturnal blood pressure, sympathetic nerve activity, and neurohormonal pathway modulation encompassing aldosterone secretion (de Souza et al. [25]; Lloberes et al. [22]).

Although the significance of BP decrease was noted by many across numerous research studies, a widespread trend that favoured CPAP medication was found [39]. Examples include Joieux-Faure et al. [26] and Fernandes et al. [30], who reported the largest, which demonstrates the importance of long-time-period remedy adherence, and Cardoso et al. [28] and Pleava et al. [27], who found further marginal improvements, which can probably be attributed to shortening of the adherence periods or lessening of adherence charges. Nocturnal blood strain reduction, aorta stiffening, other parameters of the cardiovascular system, and the heart rate were also positively stimulated by the use of CPAP in a range of studies (Cardoso et al. [28]; Navarro-Soriano et al. [29]).

The difference that has been set up during the research is, most likely, due to the differences between taking a look at structure, CPAP compliance, baseline severity of OSA, intervention period, and BP size approaches. Despite this difference, a general analysis of the data demonstrates a small and clinically significant effect of CPAP therapy on BP [40]. These findings demonstrate the significance of person-specific matters, namely, adherence to remedies and timing in the maximisation of therapeutic outcomes. In addition, consistency in benefits between the short-term and long-term period studies favours CPAP therapy as a major intervention in the management of RH in OSA patients.

Limitations

Although CPAP therapy was fully assessed concerning BP in RH and OSA patients, a number of limitations must be mentioned. First, the studies included were highly heterogeneous (I² = 92.72%), probably because of the variations in the study design, compliance with CPAP, duration of the intervention, severity of baseline OSA, and BP measurement procedures. Second, while most studies were randomised controlled trials, some were observational or prospective intervention studies, which may introduce confounding factors and reduce the certainty of causal inference. Third, the sample sizes varied considerably across studies, ranging from 33 to 194 participants, which may influence the weighting in the meta-analysis and affect the precision of pooled effect estimates. Fourth, CPAP adherence was inconsistently reported, limiting the ability to evaluate dose-response relationships and the impact of therapy compliance on outcomes. Fifth, baseline antihypertensive therapy and other co-interventions were not uniformly described, which could confound the observed effects of CPAP. Finally, the generalisability of the findings may be limited, as most studies were conducted in specific clinical settings with selected patient populations. While publication bias was not detected, the potential for unreported negative studies remains.

Future research

Although the evidence of the efficacy of CPAP therapy in lowering BP in patients with RH and OSA is consistently strong, several gaps still exist that require further research. The next research directions should focus on carrying out large-scale and multicentre randomised controlled trials to enhance the generalisability of the results and make more accurate estimates of the effect of CPAP in diverse populations. CPAP adherence monitoring must be standardised because studies have mixed adherence levels, which may influence the magnitude of BP reduction and create heterogeneity. The dose-response relationship between CPAP length of use and BP response needs to be studied, including short-term versus long-term therapy, to determine which treatment regimen would exert the largest cardiovascular effect. There is also little investigation on the interaction effect between baseline antihypertensive treatment and CPAP efficacy; future studies should endeavour to control or stratify the use of antihypertensive medication so that the effect of CPAP can be isolated. Other areas of interest to be explored are the mechanistic processes by which CPAP reduces BP, e.g., sympathetic nervous system activation, endothelial dysfunction, and neurohormonal control. Reductions in BP should be assessed with long-term follow-up studies of the hard cardiovascular events of stroke, myocardial infarction, and heart failure. Finally, research on patient-specific predictors of response to CPAP therapy is required to individualise interventions and encourage adherence, which ultimately leads to improved clinical outcomes in the high-risk group.

## Conclusions

This systematic review and meta-analysis provide comprehensive evidence on the effects of CPAP therapy on BP control in patients with RH and OSA. Across 10 included studies, CPAP consistently demonstrated beneficial reductions in both systolic and diastolic BP, with larger effects observed in patients with long-term therapy adherence. Secondary cardiovascular outcomes, including nocturnal BP dipping, heart rate, aldosterone levels, and aortic stiffness, also improved in several studies, highlighting the broader cardiovascular benefits of CPAP beyond BP reduction. Although high heterogeneity was observed among the studies, the overall trend strongly supports CPAP therapy as an effective adjunctive intervention in this high-risk population. Subgroup analyses suggest that longer treatment duration and higher adherence may enhance the magnitude of benefit, but the direction of effect remains consistently favourable across all groups. Risk of bias assessment and publication bias evaluation indicate that the results are robust and reliable, despite minor methodological variations in a few studies. In clinical practice, these findings reinforce the importance of early diagnosis and consistent CPAP use in patients with RH and OSA. Optimising adherence and individualising treatment may maximise cardiovascular benefit. Future research should focus on identifying predictors of response, standardising adherence monitoring, and assessing long-term cardiovascular outcomes to further strengthen clinical recommendations for CPAP therapy in this population.
